# Functional connectivity predicts the dispositional use of expressive suppression but not cognitive reappraisal

**DOI:** 10.1002/brb3.1493

**Published:** 2020-01-13

**Authors:** Daisy A. Burr, Tracy d'Arbeloff, Maxwell L. Elliott, Annchen R. Knodt, Bartholomew D. Brigidi, Ahmad R. Hariri

**Affiliations:** ^1^ Department of Psychology & Neuroscience Duke University Durham NC USA

**Keywords:** biomarker, emotion regulation, functional connectivity, predictive modeling

## Abstract

**Introduction:**

Previous research has identified specific brain regions associated with regulating emotion using common strategies such as expressive suppression and cognitive reappraisal. However, most research focuses on a priori regions and directs participants how to regulate, which may not reflect how people naturally regulate outside the laboratory.

**Method:**

Here, we used a data‐driven approach to investigate how individual differences in distributed intrinsic functional brain connectivity predict emotion regulation tendency outside the laboratory. Specifically, we used connectome‐based predictive modeling to extract functional connections in the brain significantly related to the dispositional use of suppression and reappraisal. These edges were then used in a predictive model and cross‐validated in novel participants to identify a neural signature that reflects individual differences in the tendency to suppress and reappraise emotion.

**Results:**

We found a significant neural signature for the dispositional use of suppression, but not reappraisal. Within this whole‐brain signature, the intrinsic connectivity of the default mode network was most informative of suppression tendency. In addition, the predictive performance of this model was significant in males, but not females.

**Conclusion:**

These findings help inform how whole‐brain networks of functional connectivity characterize how people tend to regulate emotion outside the laboratory.

## Graphical abstract

Here, we used a data‐driven approach to investigate how individual differences in distributed intrinsic functional brain connectivity predict emotion regulation tendency. We found a significant neuromarker of dispositional use of suppression, but not reappraisal. These findings help inform how whole‐brain networks of functional connectivity characterize how people tend to regulate emotion outside the laboratory.

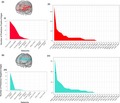

## 1 INTRODUCTION

Individuals choose to regulate their emotions in response to stressors in a variety of ways. Two common emotion regulation strategies are cognitive reappraisal and expressive suppression (Gross, 2002). Expressive suppression is an avoidance‐based regulation strategy characterized by masking outward emotional responses (Gross, 2002; Gross & John, 1998). Conversely, cognitive reappraisal involves reframing the meaning of a stimulus to change the associated emotional response (Buhle et al., 2013; Gross & John, 1998).

A major goal of emotion regulation research has been to identify how individuals vary in their tendency to suppress and reappraise. For example, research has illustrated that increased use of suppression is associated with negative health outcomes, such as anxiety (Amstadter, 2008; Cisler & Olatunji, 2012; Gross, 2002; Gross & Levenson, 1997; Troy, Wilhelm, Shallcross, & Mauss, 2010), though increased use of reappraisal is associated with lower levels of anxiety (Denny, Inhoff, Zerubavel, Davachi, & Ochsner, 2015; Jamieson, Mendes, Blackstock, & Schmader, 2010). In addition, research has demonstrated sex differences in the dispositional use of suppression and reappraisal. Women report using a wider range of regulation strategies than men (Aldao & Nolen‐Hoeksema, 2013; Nolen‐Hoeksema & Aldao, 2011) and men report using suppression more than reappraisal (Gross & John, 2003).

Emotion regulation research has aimed to characterize biomarkers associated with suppression and reappraisal (Cutuli, 2014; Dennis & Hajcak, 2009; Gross & John, 2003; Kalisch, Wiech, Herrmann, & Dolan, 2006; Kanske, Heissler, Schönfelder, & Wessa, 2012; Urry et al., 2006). For example, studies have linked reappraisal with increased activity in the dorsolateral and ventromedial prefrontal cortices (Buhle et al., 2013; Goldin, McRae, Ramel, & Gross, 2008), and both reappraisal and suppression have been associated with decreased activity in the amygdala (Buhle et al., 2013; Chen, Chen, Yang, & Yuan, 2017). Similarly, research has identified how patterns of intrinsic functional connectivity differ between suppression and reappraisal. Decreased coupling between the amygdala and medial prefrontal cortex has been associated with reappraisal, whereas increased coupling between the amygdala and dorsal anterior cingulate cortex has been associated with suppression (Pan et al., 2018; Picó‐Pérez et al., 2018; Uchida et al., 2015). Globally, intrinsic activity within the default mode network, which supports self‐referential processes, has been associated with suppression (Pan et al., 2018) and reappraisal (Gao, Chen, Biswal, Lei, & Yuan, 2018; Martins & Mather, 2016; Sripada et al., 2013; Xie et al., 2016).

Although these findings have informed the brain basis of suppression and reappraisal, they have been limited in several key areas. First, most emotion regulation research often relies on instructing individuals to regulate in specific ways (Buhle et al., 2013; McRae et al., 2010; McRae, Misra, Prasad, Pereira, & Gross, 2012). However, this may not reflect how individuals tend to regulate outside the laboratory. Second, existing studies have largely focused on a priori regions of interest, though evidence has rapidly accrued that complex cognitive processes are more likely supported by distributed networks of brain regions (Chang, Gianaros, Manuck, Krishnan, & Wager, 2015; Pan et al., 2018). Third, existing research that explores whole‐brain maps of cognitive processes is often subject to extreme issues of multiple comparisons and based on overfit models (Shen et al., 2017). Fourth, most existing studies have smaller sample sizes and amounts of data, which limits reliability and the ability to map individual differences on to the dispositional use of suppression and reappraisal (Bennett & Miller, 2010; Elliott et al., 2019).

Here, we attempt to address these limitations by using a data‐driven approach to identify patterns of distributed intrinsic functional connectivity predictive of dispositional use of suppression and reappraisal. In order to increase reliability and benefit from the most amount of data, we collapsed across task and resting‐state scans into general functional connectivity (GFC; Elliott et al., 2019). We employed connectome‐based predictive modeling (CPM; Shen et al., 2017) to select the most informative features (functional connections) from GFC matrices and predict individual differences in the dispositional use of suppression and reappraisal without overfitting the data. In light of the pronounced sex differences in the dispositional use of suppression and reappraisal (Aldao & Nolen‐Hoeksema, 2013; Gross & John, 2003; Nolen‐Hoeksema & Aldao, 2011), we examined how the predictive performance of this model varied based on sex.

## 2 METHODS

### 2.1 Participants

Data were available from 1,316 participants (age range = 18–22 years old; 43% men) who completed the Duke Neurogenetics Study between January 2010 and July 2014 (Table 1). This study was approved by the Duke University Medical Center Institutional Review Board. The authors assert that all procedures contributing to this work also complied with the ethical standards of the relevant national and institutional committees on human experimentation and with the Helsinki Declaration of 1975, as revised in 2008.

All participants provided informed consent before participation and were excluded in the present sample if they met any of the following criteria: (a) medical diagnoses of cancer, stroke, diabetes requiring insulin treatment, chronic kidney or liver disease, or lifetime history of psychotic symptoms, (b) use of psychotropic, glucocorticoid, or hypolipidemic medication, (c) conditions affecting cerebral blood flow and metabolism (e.g., hypertension), or (d) failed quality control criteria for functional magnetic resonance imaging (fMRI) data. Diagnosis of any past or current DSM‐IV Axis I disorder or select Axis II disorders (antisocial personality disorder and borderline personality disorder) were assessed with structured clinical interviews (Sheehan et al., 1998). Such diagnoses were, however, not exclusion criteria, as the Duke Neurogenetics Study sought to establish broad variability in multiple behavioral phenotypes related to psychopathology. Of the 1,316 participants included in our analyses, 66 met criteria for major depressive disorder (MDD), 35 for bipolar disorder, 26 for panic disorder, 12 for social anxiety disorder, 24 for generalized anxiety disorder (GAD), 15 for obsessive compulsive disorder (OCD), two for post‐traumatic stress disorder (PTSD), 142 for alcohol abuse, 48 for substance abuse, 11 for eating disorder (bulimia or anorexia), and three experienced psychotic symptoms.

### 2.2 Self‐report questionnaires

Individual differences in dispositional emotion regulation practices and negative affect were assessed with the following self‐report questionnaires. The Emotion Regulation Questionnaire (ERQ), a 10‐item self‐report questionnaire, was used to measure individual differences in suppression and reappraisal (Gross & John, 2003). The ERQ has two subscales—ERQ‐Suppression, including items such as “I control my emotions by not expressing them.” and ERQ‐Reappraisal, including items such as “I control my emotions by changing the way I think about the situation I'm in.” All items are rated on a 7‐point scale from 1 (strongly disagree) to 7 (strongly agree) and summed within each subscale to generate overall scores for suppression and reappraisal. The ERQ has been consistently shown to be a valid and reliable index of regulation tendency (Gross & John, 2003).

**Table 1:** Participant demographics

	Total (*N* = 1,316)	Women (*n* = 755)	Men (*n* = 561)
Age (years)	19.70 ± 1.25	19.66 ± 1.23	19.75 ± 1.27
ERQ Reappraisal (1–7)	5.18 ± 0.89	5.26 ± 0.85	5.07 ± 0.92
ERQ Suppression (1–7)	3.79 ± 1.16	3.63 ± 1.16	4.01 ± 1.13
Any diagnoses (*n*)	268	134	134
MDD (*n*)	66	44	22
Bipolar disorder (*n*)	35	19	16
Panic disorder (*n*)	26	20	6
Social anxiety disorder (*n*)	12	5	7
GAD (*n*)	24	15	9
OCD (*n*)	15	7	8
PTSD (*n*)	2	1	1
Alcohol abuse (*n*)	142	61	81
Substance abuse (*n*)	48	21	27
Eating disorder (*n*)	11	8	3
Scanner			
Scanner 1 (*n*)	1,089	625	464
Scanner 2 (*n*)	227	130	97

### 2.3 MRI data acquisition

Each participant was scanned using one of two identical research‐dedicated GE MR750 3T scanners equipped with high‐power high‐duty‐cycle 50‐mT/m gradients at 200 T m^‐1^ s^‐1^ slew rate, and an eight‐channel head coil for parallel imaging at high bandwidth up to 1 MHz at the Duke‐UNC Brain Imaging and Analysis Center. A semi‐automated high‐order shimming program was used to ensure global field homogeneity. A series of 34 interleaved axial functional slices aligned with the anterior commissure–posterior commissure plane were acquired for full‐brain coverage using an inverse‐spiral pulse sequence to reduce susceptibility artifacts (TR/TE/flip angle = 2,000 ms/30 ms/60; FOV = 240 mm; 3.75 × 3.75 × 4 mm voxels; interslice skip = 0). Four initial radiofrequency excitations were performed (and discarded) to achieve steady‐state equilibrium. For each participant, functional MRI was collected during various combinations of a single resting‐state and four task scans. Due to the multiphasic nature of the study, while all participants completed some fMRI scanning, not all participants had the same fMRI scans. See Figure S1 for a breakdown of the specific scans available for each participant.

### 2.4 MRI preprocessing

Anatomical images for each participant were skull‐stripped, intensity‐normalized, and nonlinearly warped to a study‐specific average template in the standard stereotactic space of the Montreal Neurological Institute template using the ANTs SyN registration algorithm (Avants, Epstein, Grossman, & Gee, 2008; Klein et al., 2009). Time‐series images for each participant were despiked, slice‐time‐corrected, realigned to the first volume in the time series to correct for head motion using AFNI tools (Cox, 1996), coregistered to the anatomical image using FSL's Boundary Based Registration (Cox, 1996; Greve & Fischl, 2009), spatially normalized into MNI space using the nonlinear ANTs SyN warp from the anatomical image, resampled to 2 mm isotropic voxels, and smoothed to minimize noise and residual difference in gyral anatomy with a Gaussian filter set at 6‐mm full‐width at half‐maximum. All transformations were concatenated so that a single interpolation was performed.

Time‐series images for each participant were furthered processed to limit the influence of motion and other artifacts. Voxel‐wise signal intensities were scaled to yield a time series mean of 100 for each voxel. Motion regressors were created using each participant's six motion correction parameters (three rotation and three translation) and their first derivatives (Jo et al., 2013; Satterthwaite et al., 2013) yielding 12 motion regressors. White matter and cerebrospinal fluid nuisance regressors were created using CompCorr (Behzadi, Restom, Liau, & Liu, 2007). Images were bandpass filtered to retain frequencies between 0.008 and 0.1 Hz, and volumes exceeding 0.25 mm frame‐wise displacement or 1.55 standardized DVARS (Nichols, 2017; Power et al., 2014) were censored. Nuisance regression, bandpass filtering, and censoring for each time series were performed in a single processing step using AFNI's 3dTproject.

### 2.5 General functional connectivity

We combined all available BOLD data (task and resting‐state) for each participant into a single time series. Using our recently developed methods (Elliott et al., 2019), we extracted measures of GFC for each participant using a 264‐region parcellation scheme derived in a large independent dataset (Power et al., 2011). BOLD time series were averaged within 5‐mm spheres surrounding each of the 264 coordinates in the parcellation and extracted time series were concatenated. Importantly, we regressed out the task structure from each time series to reduce the effect of task‐related activation on estimates of functional connectivity (Elliott et al., 2019). Correlation matrices were derived from these time series using Pearson correlation, resulting in 34,716 edges in the Power et al. parcellation. Sensitivity analyses excluding participants with fewer than 400 TRs (Elliott et al., 2019) did not change our findings reported below.

### 2.6 Connectome‐based predictive modeling

Dispositional use of suppression and reappraisal was independently predicted from patterns of GFC using CPM (Shen et al., 2017). This framework provides a general method to predict any measure from intrinsic connectivity matrices. Functional connections in the brain that had a *p* < .01 correlation with self‐reported suppression and reappraise tendency were selected and used as features in a predictive model. Three linear regression predictive models were then built—one from the positive features (edges positively correlated with the measure of interest), one from the negative features (edges negatively correlated with the measure of interest) and one from the combination of positive and negative features (Shen et al., 2017). Here, we discuss the combined model that predicts dispositional regulation styles from positive and negative features in the brain (Shen et al., 2017).

Models were trained using a leave‐one‐out cross‐validation scheme wherein data from all participants except one were used to predict the measure in the left‐out participant. This was repeated until all participants had been left out. The Spearman correlation between predicted and true scores was adopted as an unbiased effect size measure of predictive utility. Model predictions of suppression and reappraisal tendency were assessed for significance using a parametric test for significance of correlations. All *p* values from correlations with suppression and reappraisal tendency were corrected for multiple comparisons using the false discovery rate (Benjamini & Hochberg, 1995). All confidence intervals for CPM prediction estimates were generated with bootstrap resampling, using AFNI's 1dCorrelate tool.

To first establish and then separate between‐ and within‐network GFC, significant positive and negative edges for each of the 264 nodes were independently sorted into seven established neural networks (Yeo et al., 2011) based on predefined network assignations of each node. Any node that did not fall into one of these established networks was assigned to an “other” category. The number of edges within and between networks was then assessed for significance using random‐sorted permutation testing to establish null distributions for comparison and determine how many connections would be expected by chance (*p* < .001; after correcting for multiple comparisons [0.05/38 total within and between comparisons = 0.001]). In order to examine sex differences in the predictive performance of the models, we correlated the true and predicted dispositional regulation measures (i.e., the output of the CPM model) separately for males and females. Based on previous behavioral and neuroimaging literature noting that men use suppression more than females (Aldao & Nolen‐Hoeksema, 2013; Cai, Lou, Long, & Yuan, 2016; Gross & John, 2003; McRae, Ochsner, Mauss, Gabrieli, & Gross, 2008; Nolen‐Hoeksema & Aldao, 2011), we predicted that this correlation would be stronger in males.

## 3 RESULTS

### 3.1 Self‐report measures

Means and standard deviations for the self‐report questionnaires were as follows: ERQ‐Suppression (3.79 ± 1.16, range = 7) and ERQ‐Reappraisal (5.18 ± 0.89, range = 7). There were significant sex differences (*t*(1223.7) = 6.05, *p* < .001) in ERQ‐Suppression subscores, with men (4.01 ± 1.13) scoring higher than women (3.63 ± 1.16). There were significant sex differences (*t*(1159.2) = −3.78, *p* < .001) in ERQ‐Reappraisal subscale scores as well, with women (5.26 ± 0.86) scoring higher than men (5.07 ± 0.92). ERQ‐Reappraisal and ERQ‐Suppression subscales were not significantly correlated (*r* = −.04, *p* = .97).

### 3.2 Connectome‐based predictive modeling

**Figure 1:**
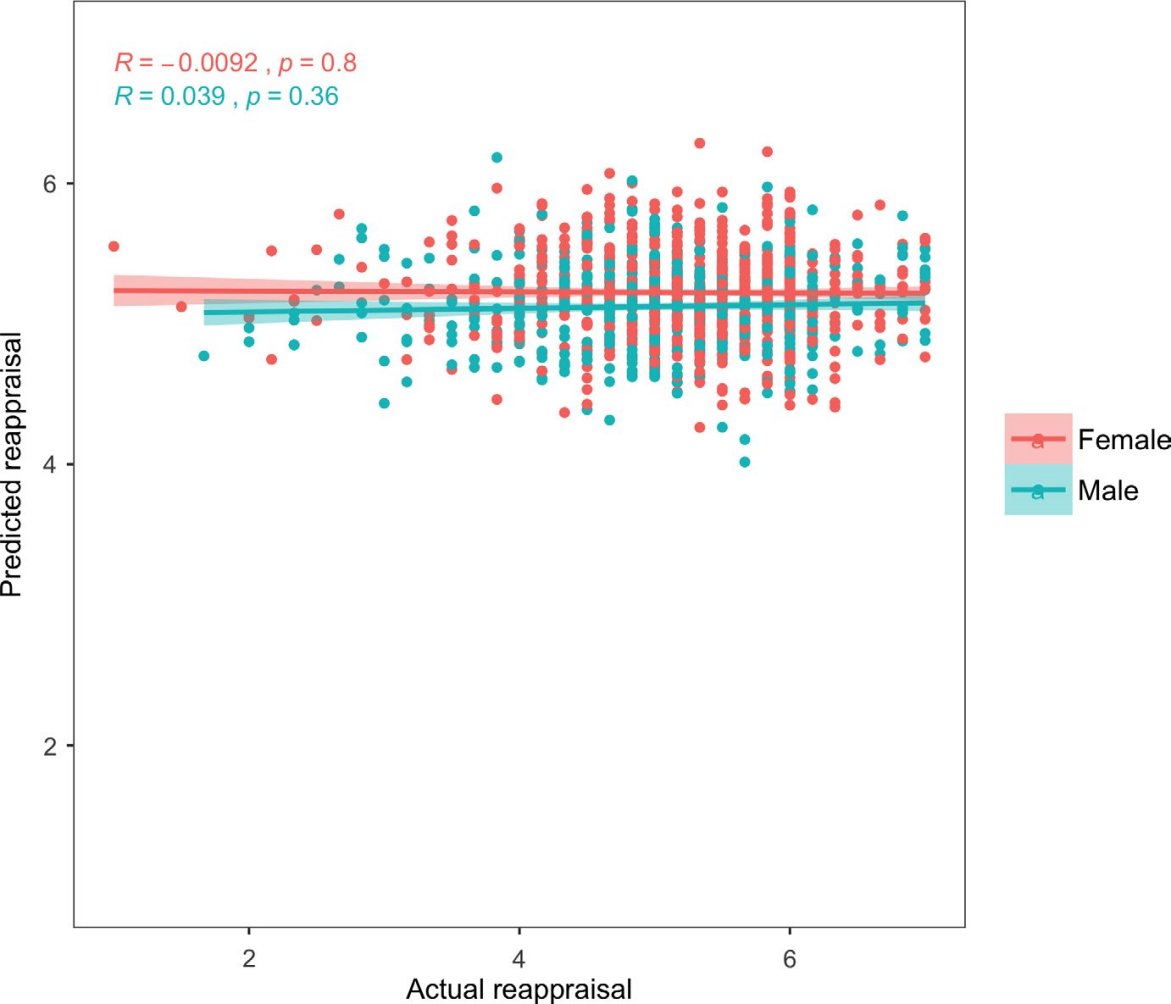
Correlation between actual and predicted Emotion Regulation Questionnaire‐Reappraisal subscale scores from the connectome‐based predictive model (*r*_male_ = .039, *p* = .36; *r*_female_ = −.009, *p* = .8)

We investigated whether there was a whole‐brain signature for the dispositional use of suppression and reappraisal using a predictive and cross‐validated model. There was no pattern of distributed functional connectivity in the brain that predicted individual differences in the dispositional use of reappraisal (*r* = .02, *p* = .37). We compared the correlations between actual and predicted ERQ‐Reappraisal scores (i.e., the output and predictive utility of the CPM models) in men and women and found no sex difference (*r*_male_ = −.009, *p* = .8; *r*_female_ = .039, *p* = .36; Figure 1).

**Figure 2:**
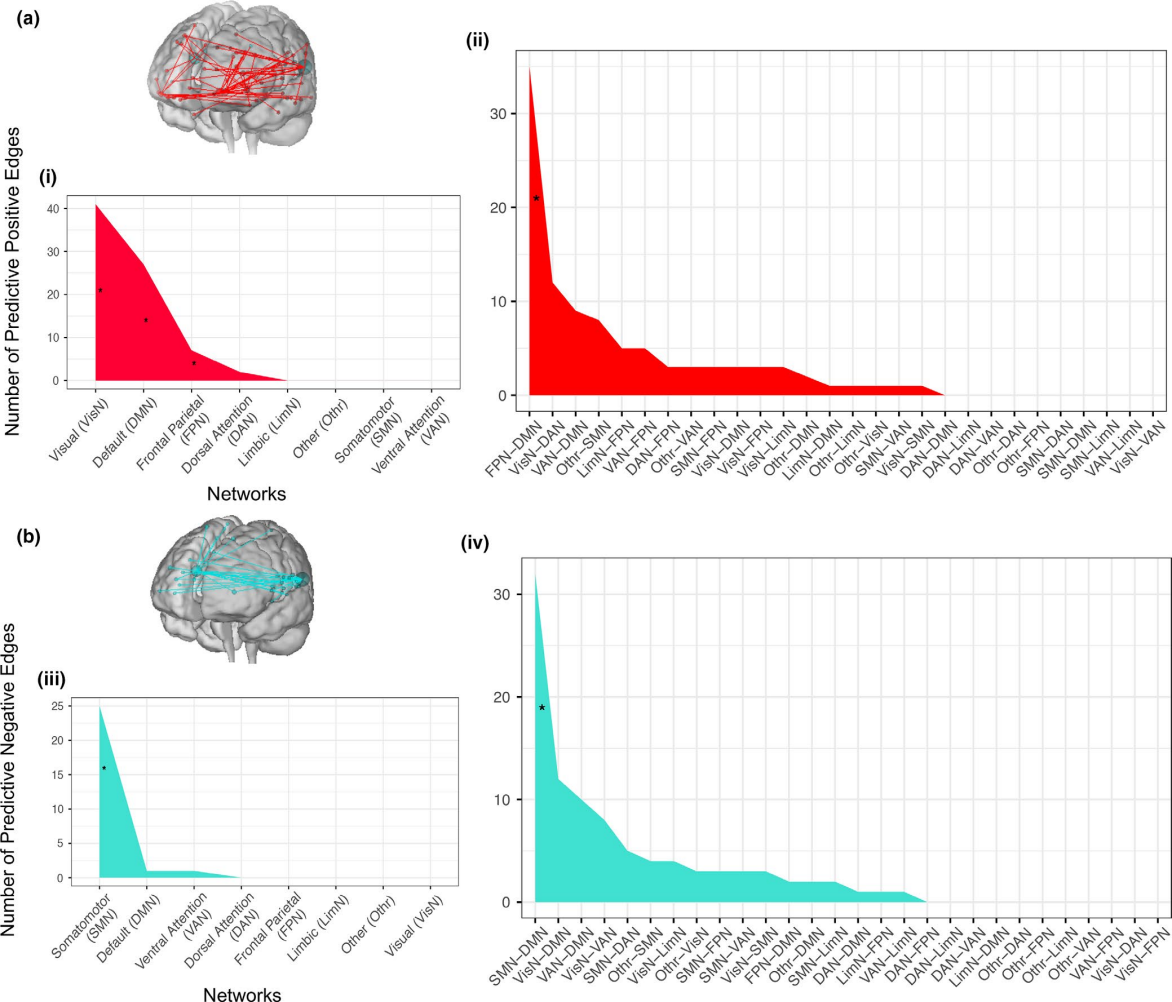
(a) The number of predictive positive edges within each network (i) and the number of predictive positive edges between each network that predict typical use of expressive suppression (ii). (b) The number of predictive negative edges within each network (iii) and the number of predictive negative edges between each network that predict typical use of expressive suppression (iv). *Denotes networks that are significantly (*p* < .001; based on Bonferroni multiple comparisons corrections) above the null distribution. Glass brain figures created by http://bisweb.yale.edu/connviewer/ (Shen et al., 2017)

In contrast, there was a whole‐brain signature for the dispositional use of suppression (*r* = .135, *p* < .001). A total of 176 functional connections were positively correlated and 123 functional connections were negatively correlated with ERQ‐Suppression (Table 2). Within‐network analyses revealed that the visual network had the most (41) positive predictive edges (Figure 2a) and the somatomotor network had the most (25) negative predictive edges (Figure 2b). The most (35) between‐network positive connections were across frontoparietal and default mode networks, and the most (32) between‐network negative connections were across somatomotor and default mode networks (Figure 2a,b). Random sampling permutation tests of within‐network patterns confirmed significant positive associations for visual, default mode, and frontoparietal networks, and significant negative associations for the somatomotor network. The only between‐network connections expected above chance were across default mode and frontoparietal networks (positive) and default mode and somatomotor networks (negative).

**Table 2:** Predictive edges

Network	Direction	
	Positive	Negative
Within		
Visual (VisN)	41*	0
Default mode (DMN)	27*	1
Frontoparietal (FPN)	7*	0
Dorsal attention (DAN)	2	0
Somatomotor (SMN)	0	25*
Ventral attention (VAN)	0	1
Limbic (LimN)	0	0
Other (Othr)	0	0
Between		
FPN ‐ DMN	35*	2
VisN ‐ DAN	12	0
VAN ‐ DMN	9	10
Othr ‐ SMN	8	4
LimN ‐ FPN	5	1
VAN ‐ FPN	5	0
VisN ‐ DMN	3	12
VisN ‐ LimN	3	4
SMN ‐ FPN	3	3
DAN ‐ FPN	3	0
Othr ‐ VAN	3	0
VisN ‐ FPN	3	0
Othr ‐ DMN	2	2
Othr ‐ VisN	1	3
SMN ‐ VAN	1	3
VisN ‐ SMN	1	3
LimN ‐ DMN	1	0
Othr ‐ LimN	1	0
SMN ‐ DMN	0	32*
VisN ‐ VAN	0	8
SMN ‐ DAN	0	5
SMN ‐ LimN	0	2
DAN ‐ DMN	0	1
VAN ‐ LimN	0	1
DAN ‐ LimN	0	0
DAN ‐ VAN	0	0
Othr ‐ DAN	0	0
Othr ‐ FPN	0	0
Total	176	123

* Denotes networks that are significantly (*p* <. 001; based on Bonferroni multiple comparisons correction) above the null distritbuion.

**Figure 3:**
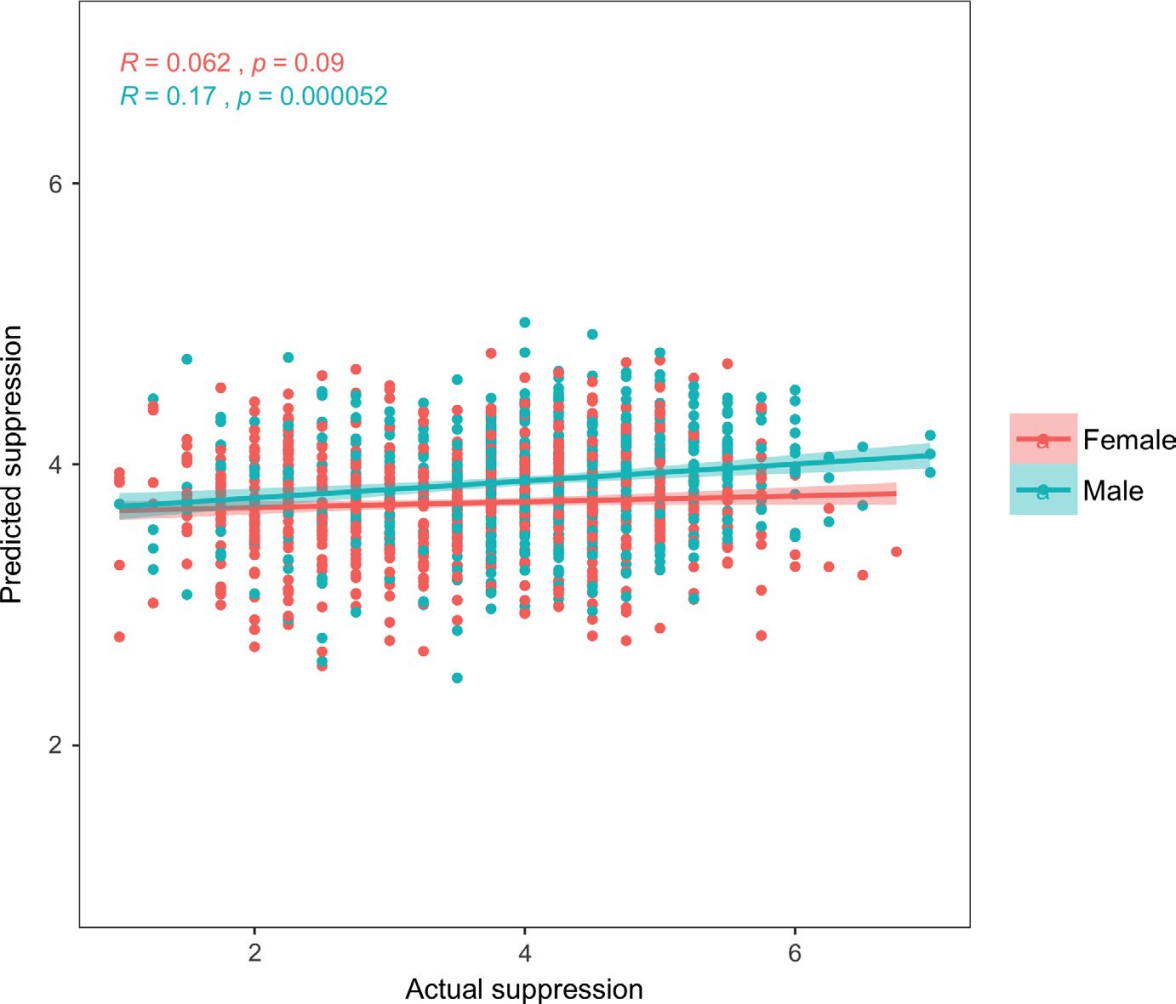
Correlation between actual and predicted Emotion Regulation Questionnaire‐Suppression subscale scores from the connectome‐based predictive model (*r*_male_ = .17, *p* < .001; *r*_female_ = .062, *p* = .09)

When examining sex differences in the neural signature of dispositional suppression, we found that the predictive performance of the model (the correlation between actual and predicted ERQ‐Suppression scores) was only significant in men (*r*_male_ = .17, *p* < .001; *r*_female_ = .05, *p* = .09; Figure 3). Moreover, the correlation coefficients between men and women were significantly different (*z* = 1.96, *p* = .049). In addition, we reran our CPM model with past or present psychiatric diagnosis as a covariate, which confirmed the neural signature associated with suppression was not driven by diagnosis (*r* = .13,* p* < .001). Because not all data were collected on the same scanner (Table 1), we conducted a final sensitivity analysis included scanner as a covariate, which confirmed the neural signature associated with suppression was not driven by scanner differences (*r* = .11, *p* < .001).

## 4 DISCUSSION

In a large sample of 1,316 participants, we used a data‐driven and theory‐free approach to examine whether there is a neural signature for how people tend to regulate their emotions. Instead of limiting patterns of functional connectivity to the typical 5–10 min of resting‐state scans (Elliott et al., 2019), we used GFC to leverage shared features of task and resting‐state fMRI and generate more reliable estimates of intrinsic functional connectivity. Most available research investigating individual differences in intrinsic connectivity is based on resting‐state scans. However, most resting‐state scans are typically not sufficiently long enough to generate reliable estimates of intrinsic functional connectivity. GFC has been shown to be a reliable measure of stable, trait‐like individual differences in behavior, such as dispositional regulation tendency (Elliott et al., 2019). By adopting GFC, we increased our ability to investigate neural signatures of individual differences in emotion regulation tendency.

In addition to benefiting from using all available functional data, our findings begin to address other limitations often present in emotion regulation research. For example, existing research often finds overlapping patterns of neural activity for suppression and reappraisal, confounding the ability to distinguish between them. As our approach is wholly data‐driven, we are able to analyze distributed patterns for each strategy separately. In addition, existing neuroimaging research that explores brain‐behavior relationships is often biased toward specific regions of interest or explores whole‐brain maps that may overfit and be vulnerable to false positives (Shen et al., 2017). Using CPM, we circumvent these limitations and generate a predictive model that is cross‐validated in novel samples.

As with prior research on whole‐brain networks associated with suppression, our results indicate that functional connectivity between the frontoparietal and default mode networks is positively correlated with the tendency to suppress negative emotion (Pan et al., 2018). The default mode network, initially named so because it is active in the absence of an explicit goal or task, is commonly implicated in self‐reflection, mind wandering, and self‐generated thought (Andrews‐Hanna, 2012; Andrews‐Hanna, Smallwood, & Spreng, 2014). The default mode network has been implicated in suppression in prior studies employing a univariate analysis approach as well (Goldin et al., 2008). This dual recruitment of default mode and frontoparietal networks is consistent with prior research indicating that the default mode network may support emotional processing and reflection, and the frontoparietal network may support emotional control—abilities that align to primary descriptions of suppression as the conscious inhibition of emotional expression (Gross & Levenson, 1993).

Consistent with demonstrated sex differences in emotion regulation, the neural signature of dispositional use of suppression identified in our current study was specific to men who tend to habitually use and are more successful at implementing suppression (Cai et al., 2016; Gross & John, 2003). Similarly, electromyography findings have shown that men show reduced emotional expressions when viewing negative emotional stimuli (Grossman & Wood, 1993). Prior research has shown that men are socialized to refrain from expressing emotion and therefore have more developmental experience with suppressing negative emotion (Brody & Hall, 2010; Katkin & Hoffman, 1976; Williams & Best, 1990). Such differences may contribute to our sex‐specific effects. Future work is necessary to replicate this sex difference.

Importantly, the neural signature of dispositional suppression remained significant after excluding participants with psychiatric diagnoses, suggesting that clinical manifestations associated with suppressing emotion are not driving the predictive relationship between patterns of functional connectivity and suppression tendency. Although the tendency to suppress negative emotion is one characteristic of depression and anxiety (Amstadter, 2008; Cisler & Olatunji, 2012; Gross, 2002; Gross & Levenson, 1997; Troy et al., 2010), healthy adults may also use suppression in certain situations (Doré, Silvers, & Ochsner, 2016; Suri et al., 2018). Thus, the use of suppression is likely not categorically maladaptive and, indeed, may be adaptive in particularly stressful situations (Doré et al., 2016).

We found a pronounced whole‐brain signature of the tendency to suppress negative emotion, but found no comparable signature for reappraisal. Specifically, despite prior research implicating the default mode network in the reappraisal of negative emotion, we found no such signature. However, these findings typically come from studies with small sample sizes, which increase the likelihood of false positives (Gao et al., 2018). Moreover, emotion regulation research typically involves instructing participants to employ an explicit strategy (Cutuli, 2014; Dennis & Hajcak, 2009; Gross & John, 2003; Kalisch et al., 2006; Kanske et al., 2012; Urry et al., 2006). But how individuals regulate in the laboratory may not reflect how they regulate in the real world (Aldao, Nolen‐Hoeksema, & Schweizer, 2010; Gross & John, 2003; Moore, Zoellner, & Mollenholt, 2008).

One possibility for not identifying a neural signature for reappraisal could be that reappraisal is a more heterogeneous construct with numerous subtypes than suppression (Doré et al., 2016). For example, although reappraisal is predominantly thought to be implemented in the service of reframing a stimulus to be less negative, individuals may use reappraisal to reframe a stimulus to be more positive (Doré et al., 2016). In addition, people can reappraise stimuli to feel less personal and more detached (Ossenfort, Harris, Platzek, & Isaacowtiz, 2018). In contrast, suppression is a more homogenous strategy with less variability in how it can be implemented. As opposed to having numerous possible goals and subtypes, expressive suppression is a less complicated technique that invariably involves trying to mask your feelings from the outside world (Gross, 2002; Gross & John, 1998). Therefore, it may be more difficult to capture a stable neural signature of something as variable and multifaceted as reappraisal in comparison with suppression. Relatedly, the behavioral phenotypes predicted by our models are based on the ERQ‐Suppression and ERQ‐Reappraisal subscales. The heterogeneity of reappraisal may be adding too much noise for the scale to be significantly predicted from patterns of functional connectivity. Relatedly, it may be difficult to self‐report on a cognitively demanding and multifaceted technique such as reappraisal, making it difficult for a brain–behavior relationship to be identified.

Our study is not without limitations. Although GFC explicitly removes task‐related activation from estimates of functional connectivity, such connectivity may nevertheless be affected by the inherent task performed during data acquisition (Elliott et al., 2019). Future research could investigate how functional connectivity estimated only from resting‐state data maps onto the dispositional use of reappraisal and suppression. Future studies may also aim to include a more diverse sample, as our sample was comprised of relatively high‐functioning university students. Similarly, our data were cross‐sectional, which limited our ability to determine whether GFC patterns drive the use of suppression or whether the use of suppression drives changes in GFC. Lastly, the ERQ measures the tendency to use either suppression or reappraisal, but does not capture alternative strategies that people may adopt.

## CONFLICT OF INTEREST

The authors declare no competing financial interests.

## Data Availability

All data are available upon request.
