# Supplementary material for: Functional connectivity predicts the dispositional use of expressive suppression but not cognitive reappraisal
Source: Brain Behav. 2020 Jan 13;10(2):e01493. doi: 10.1002/brb3.1493 (PMC7010583; doi:10.1002/brb3.1493)
Supplement: Supplementary file 1 [file BRB3-10-e01493-s001.docx]

**Supplementary Figures**

| Amygdala Reactivity Task | Ventral Striatum  Task | Hippocampal  Task | Working Memory Task | Resting State  Scan | # Subjects |
| --- | --- | --- | --- | --- | --- |
|  |  |  |  |  | 960 |
|  |  |  |  |  | 306 |
|  |  |  |  |  | 29 |
|  |  |  |  |  | 8 |
|  |  |  |  |  | 3 |
|  |  |  |  |  | 3 |
|  |  |  |  |  | 2 |
|  |  |  |  |  | 2 |
|  |  |  |  |  | 1 |
|  |  |  |  |  | 1 |
|  |  |  |  |  | 1 |
|  |  |  |  | Total | 1316 |

*Figure 1.* Breakdown by task of available Magnetic Resonance Imaging data for participants (N = 1,316). Mean available Repetition Time (TRs) across all participants was 620.2 (1st Quartile = 494, 3rd Quartile = 622). Colored squares signify data availability. See Supplementary Material for detailed descriptions of each task.

**Supplementary Material**

*Amygdala Task*

The experimental fMRI paradigm consists of four blocks of a perceptual face-matching task interleaved with five blocks of a sensorimotor control task. The Duke Neurogenetics Study version of this paradigm consists of one block each of fearful, angry, surprised, and neutral facial expressions presented in a pseudorandom order across participants. During face-matching blocks, participants view a trio of faces and select one of two faces identical to a target face. Each face processing block consists of six images, balanced for gender, all of which were derived from a standard set of pictures of facial affect (Ekman & Friesen, 1976). During the sensorimotor control blocks, participants view a trio of simple geometric shapes (circles and vertical and horizontal ellipses) and select one of two shapes that are identical to a target shape. Each sensorimotor control block consists of six different shape trios. All blocks are preceded by a brief instruction ("Match Faces" or "Match Shapes") that lasts 2 s. In the task blocks, each of the six face trios is presented for 4 s with a variable interstimulus interval (ISI) of 2-6 s (mean = 4 s) for a total block length of 48 s. A variable ISI is used to minimize expectancy effects and resulting habituation, and maximize amygdala reactivity throughout the paradigm. In the control blocks, each of the six shape trios is presented for 4 s with a fixed ISI of 2 s for a total block length of 36 s. Total task time is 390 s.

*Ventral Striatum Task*

As described previously (Forbes et al. 2009), our blocked-design number-guessing paradigm consists of a pseudorandom presentation of three blocks of predominantly positive feedback (80% correct guess), three blocks of predominantly negative feedback (20% correct guess) and three control blocks. There are five trials in 3 seconds to guess, via button press, whether the value of a visually presented card is lower or higher than 5 (index and middle finger, respectively). The numerical value of the card is then presented for 500 milliseconds and followed by appropriate feedback (green upward-facing arrow for positive feedback; red downward-facing arrow for negative feedback) for an additional 500 milliseconds. A crosshair is then presented for 3 seconds, for a total trial length of 7 seconds. Each block comprises five trails, with three blocks each of predominantly positive feedback (80% correct) and three of predominantly negative feedback (20% correct) interleaved with three control blocks. During control blocks, participants are instructed to simply make button presses during the presentation of an "x" (3 seconds), which is followed by an asterisk (500 milliseconds) and a yellow circle (500 milliseconds). Each block is preceded by an instruction of "Guess Number" (positive or negative feedback blocks) or "Press Button" (control blocks) for 2 seconds resulting in a total block length of 38 seconds and a total task length of 342 seconds. Participants were unaware of the fixed outcome probabilities associated with each block and were led to believe that their performance would determine a net monetary gain at the end of the scanning session. Instead, all participants received $10. We included one incongruent trial within each task block (eg, one of five trials during positive feedback blocks was incorrect resulting in negative feedback) to prevent participants from anticipating the feedback for each trial and to maintain participants' engagement and motivation to perform well.

*Hippocampus Task*

Our fMRI paradigm consists of the encoding and subsequent recall of novel face-name pairs (Zeineh et al., 2003). A distractor task (odd/even number identification) is interleaved between encoding and recall blocks to prevent maintenance of information in working memory. During each of four encoding blocks, participants view six novel face-name pairs for 3.5 seconds each. During each of four recall blocks, participants view six faces each presented for 2 seconds and immediately followed by an incomplete name fragment for 1 second during which they are required by forced-choice to determine if the fragment is correct or incorrect. A 1 second inter-trial interval is used during recall blocks. During each of four distractor blocks, participants view six different numbers for 3.5 seconds each and are required to determine if the numbers are odd or even (see [diagram](https://www.haririlab.com/img/facename.png)). Total task length is 324 seconds.

*Prefrontal Task*

Activity of the dlPFC was measured during BOLD fMRI using an event-related working memory paradigm adapted from Tan et al. (Tan et al., 2007, Scult et al. 2016). The paradigm included 10 trials for each of 6 different conditions, including 3 control conditions, consisting only of a 3s response phase, and 3 working memory conditions, consisting of a 0.5s encoding phase followed by a 4s maintenance interval and a 3s response phase. Control and working memory conditions were interleaved with jittered rest intervals lasting 4s to 8.5s for a total scan length of 11m 48s. Responses were recorded via an MR-compatible button box using the index (left button) and middle (right button) fingers of the dominant hand.

During the control conditions, participants performed 1) a simple motor task in which they pressed either the left or the right button according to a prompt, 2) a numerical size judgment task in which they chose the number on the left or right based on an instruction to choose either the larger or the smaller number, and 3) a numerical computation and size judgment task in which they performed a numerical subtraction of 2 or 3 from either the left or right number, and made a numerical size judgment as instructed.

In the first working memory condition, participants viewed 2 numbers during the brief encoding phase, then recalled the numbers and performed a numerical size judgment as instructed. In the second working memory condition, the participants additionally performed subtraction of 2 or 3 from one of the remembered numbers as indicated before making the numerical size judgment during recall. In the final working memory condition, participants performed subtraction of 2 or 3 from one of the 2 numbers during the brief encoding phase, then recalled the resulting two numbers and performed a numerical size judgment as instructed during the response phase after the maintenance interval. In each working memory condition trial, all the numbers were single digits from 0 to 9; the two numbers on which the numerical size judgment was ultimately performed (after numerical computation if applicable) were equally balanced across 0 to 9, and equally likely to differ by either 1 or 3 units. Numerical computation was equally likely on the left or right number, with correct responses equally balanced on the left or right, and equally likely to be the larger or smaller number for each working memory trial type. The trials were performed in an order that was optimized using a sequencing program (Wager & Nichols, 2003).

**Supplementary References**

Ekman P, Friesen W. Pictures of Facial Affect. Palo Alto, CA: Consulting Psychologists Press;

1976.

Forbes, EE, Brown, SM, Kimak, M, Ferrell, RE, Manuck, SB, Hariri, AR (2009). Genetic

variation in components of dopamine neurotransmission impacts ventral striatal reactivity

associated with impulsivity. Molecular Psychiatry 14, 60-70.Scult, M. A., Knodt, A. R., Swartz, J. R., Brigidi, B. D., & Hariri, A. R. (2016). Thinking and Feeling: Individual

Differences in Habitual Emotion Regulation and Stress-Related Mood Are Associated

With Prefrontal Executive Control. Clinical Psychological Science,

2167702616654688.

Tan, H.-Y., Chen, Q., Goldberg, T. E., Mattay, V. S., Meyer-Lindenberg, A., Weinberger, D. R.,

& Callicott, J. H. (2007). Catechol-O-methyltransferase Val158Met modulation of prefrontal-parietal-striatal brain systems during arithmetic and temporal transformations

in working memory. The Journal of Neuroscience: The Official Journal of the Society for Neuroscience, 27(49), 13393-401. doi:10.1523/JNEUROSCI.4041-07.2007

Wager, T. D., & Nichols, T. E. (2003). Optimization of experimental design in fMRI: A general

framework using a genetic algorithm. NeuroImage, 18(2), 293-309. doi:10.1016/S1053-8119(02)00046-0

Zeineh MM, Engel SA, Thompson PM, Bookheimer SY. Dynamics of the hippocampus during

encoding and retrieval of face-name pairs. Science. 2003;299:577–580.
